# Does fasting plasma glucose values 5.1-5.6 mmol/l in the first trimester of gestation a matter?

**DOI:** 10.3389/fendo.2023.1155007

**Published:** 2023-06-02

**Authors:** Fahimeh Ramezani Tehrani, Farshad Farzadfar, Farhad Hosseinpanah, Maryam Rahmati, Faegheh Firouzi, Mehrandokht Abedini, Farzad Hadaegh, Majid Valizadeh, Farahnaz Torkestani, Davood Khalili, Masoud Solaymani-Dodaran, Razieh Bidhendi-Yarandi, Marzieh Bakhshandeh, Afshin Ostovar, Marzieh Rostami Dovom, Mina Amiri, Fereidoun Azizi, Samira Behboudi-Gandevani

**Affiliations:** ^1^ Reproductive Endocrinology Research Center, Research Institute for Endocrine Sciences, Shahid Beheshti University of Medical Sciences, Tehran, Iran; ^2^ Non-Communicable Diseases Research Center, Endocrinology and Metabolism Population Sciences Institute, Tehran University of Medical Sciences, Tehran, Iran; ^3^ Obesity Research Center, Research Institute for Endocrine Sciences, Shahid Beheshti University of Medical Sciences, Tehran, Iran; ^4^ Infertility and Cell Therapy Office, Transplant & Disease Treatment Center, Ministry of Health and Medical Education, Tehran, Iran; ^5^ Prevention of Metabolic Disorders Research Center, Research Institute for Endocrine Sciences, Shahid Beheshti University of Medical Sciences, Tehran, Iran; ^6^ Faculty of Medicine, Shahed University, Tehran, Iran; ^7^ Minimally Invasive Surgery Research Center, Iran University of Medical Sciences, Tehran, Iran; ^8^ Department of Biostatistics, University of Social Welfare and Rehabilitation Sciences, Tehran, Iran; ^9^ Family Health Department, Ministry of Health and Medical Education, Tehran, Iran; ^10^ Osteoporosis Research Center, Endocrinology and Metabolism Clinical Sciences Institute, Tehran University of Medical Sciences, Tehran, Iran; ^11^ Endocrine Research Center, Research Institute for Endocrine Sciences, Shahid Beheshti University of Medical Sciences, Tehran, Iran; ^12^ Faculty of Nursing and Health Sciences, Nord University, Bodø, Norway

**Keywords:** early screening, randomized non-inferiority field trial, gestational diabetes, first trimester of gestation, glucose values 5.1-5.6 mmol/l

## Abstract

**Objectives:**

The aim of the study was to investigate the effect of treatment on pregnancy outcomes among women who had fasting plasma glucose (FPG) 5.1-5.6 mmol/l in the first trimester of pregnancy.

**Methods:**

We performed a secondary-analysis of a randomized community non-inferiority trial of gestational diabetes mellitus (GDM) screening. All pregnant women with FPG values range 5.1-5.6 mmol/l in the first trimester of gestation were included in the present study (n=3297) and classified to either the (i) intervention group who received treatment for GDM along with usual prenatal care (n=1,198), (ii) control group who received usual-prenatal-care (n=2,099). Macrosomia/large for gestational age (LGA) and primary cesarean-section (C-S) were considered as primary-outcomes. A modified-Poisson-regression for binary outcome data with a log link function and robust error variance was used to RR (95%CI) for the associations between GDM status and incidence of pregnancy outcomes.

**Results:**

The mean maternal age and BMI of pregnant women in both study groups were similar. There were no statistically significant differences in the adjusted risks of adverse pregnancy outcomes, including macrosomia, primary C-S, preterm birth, hyperbilirubinemia, preeclampsia, NICU-admission, birth trauma, and LBW both groups.

**Conclusions:**

It is found that treating women with first-trimester FPG values of 5.1-5.6 mmol/l could not improve adverse pregnancy outcomes including macrosomia, Primary C-S, Preterm birth, hypoglycemia, hypocalcemia, preeclampsia, NICU admission, Birth trauma and LBW. Therefore, extrapolating the FPG cut-off point of the second trimester to the first –which has been proposed by the IADPSG, might therefore not be appropriate.

**Clinical Trial Registration:**

https://www.irct.ir/trial/518, identifier IRCT138707081281N1.

## Introduction

Gestational diabetes (GDM) is one of the most common endocrinopathies during gestation (1, 2). Traditionally, GDM screening and diagnosis have been based on oral glucose tolerance test (OGTT) in the second trimester of pregnancy (3, 4). During this time, placenta produces diabetogenic hormones such as human placental lactogen which can lead to progressive insulin resistance and elevated blood glucose levels in individuals with insufficient insulin production to maintain euglycemia (5). Likewise, the results of the Hyperglycemia and Adverse Pregnancy Outcome (HAPO) study have confirmed a continuous association between adverse pregnancy outcomes and maternal glucose levels that are that are less severe than those in overt diabetes mellitus (DM) (6).

It is widely recognized that untreated overt DM during pregnancy is strongly associated with adverse feto-maternal and neonatal outcomes (7). Due to the increasing rate of undiagnosed DM among pregnant women in early gestation, the International Association of the Diabetes and Pregnancy Study Groups (IADPSG) and the World Health Organization (WHO) have recommended first-trimester screening to identify overt diabetes in pregnancy (ODIP) (1). Early screening also identifies women with hyperglycemia that is less than ODIP (GDM). However, with limited trial data and by extrapolating results from the HAPO study conducted during the second trimester to early gestation, IADPSG has endorsed the diagnostic criteria of GDM for fasting plasma glucose (FPG) in the range of 5.1-6.9 mmol/l (1). Nevertheless, emerging data has challenged this recommendation since many such women no longer meet the criteria for GDM when later re-screened during the second trimester of pregnancy (8–13).

Meanwhile, most cases of additional early-onset GDM were identified based on a FPG of 5.1-5.6 mmol/L during the first trimester of gestation (14, 15). These stringent criteria may potentially result in a higher prevalence of GDM, without any clear evidence to reduce the risk for adverse pregnancy outcomes. Besides, it may potentially increase the costs of care and over-medicalization of pregnancy for a large number of previously healthy pregnant women who will now be labeled as patients, especially in low- and middle-income countries with limited resources (16). Furthermore, the diagnosis and treatment of GDM may be a stressful situation accompanied by serious psychological consequences, as well as impair the quality of life for women and their families (17, 18). Therefore this study aimed to investigate the effects of treatment in women who had a fasting plasma glucose (FPG) 5.1-5.6 mmol/l in the first trimester of gestation on maternal and neonatal outcomes.

## Materials and methods

This study presents a secondary analysis of a randomized community non-inferiority trial among pregnant women. Detailed description of the methods used has been reported previously (14, 19, 20). In summary, a total of 35,430 pregnant women in their first trimester were recruited from five different geographic regions of Iran across 25 selected cities. Those who were younger than 18 years, suffered from overt DM or other chronic disorders, were uncertain about their gestational age (no ultrasound estimation from 6 to 14 weeks of gestational age available and last menstrual period not certain) were excluded from the study. All pregnant women received standard prenatal care and were screened for GDM twice. The first screening was conducted in the first trimester using an FPG measurement, and the second screening was performed in the second trimester using either a one-step or a two-step screening method. Based on the GDM screening approach, participants were randomized to 5 protocols: In Protocol A, GDM was defined as a FPG between 93 mg/dL and 126 mg/dL in the first trimester, and any abnormal value using the one-step screening method in the second trimester with a 2-hour 75-gram oral glucose tolerance test (OGTT) and cutoff values of fasting 93 mg/dL, 1-hour 180 mg/dL, or 2-h 153 mg/dL. Protocol B differed from Protocol A in the definition of GDM in the first trimester, which was FPG between 100 mg/dL and 126 mg/dL, and in the second trimester, which was defined as two or more plasma glucose levels meeting or exceeding the criteria. Protocol C used the same definition for GDM in the first trimester as Protocol B (FPG between 100 mg/dL and 126mg/dL), and the same definition in the second trimester as Protocol A (any abnormal value using the one step screening method with a 2-hour, 75-gram GTT). Protocol D defined GDM in the first trimester as FPG values between 93 mg/dL and 126 mg/dL. However, in the second trimester, a two-step screening method was used, using the cut-off values of Carpenter-Coustan criteria. Protocol E differed from Protocol D in the definition of GDM in the first trimester, which was FPG between 100 mg/dL and 126 mg.

For current analysis, we restructured the original data. In this respect, those pregnant women with FPG values range 5.1-5.6 mmol/l in the first trimester of gestation were selected form original data (n=3297) and included in this secondary analysis. Then, they were classified to either the (i) intervention group who received treatment for GDM along with usual prenatal care (n=1,198), (ii) control group who received usual-prenatal-care (n=2,099). All women in the control group were re-screened for GDM between 24–28 weeks of gestation using either a one-step or a two-step screening approach. It should be noted that receiving GDM treatment in the intervention group and or usual prenatal care in controls were defined by original study randomization. On the other hand, the current study’s restructuring of the data and classification of participants into the intervention and control groups did not affect the original randomization process of the original trial. Therefore, the control group in the current study was not influenced by any new arbitrary decisions or approaches made during the secondary analysis.

**Figure 1:**
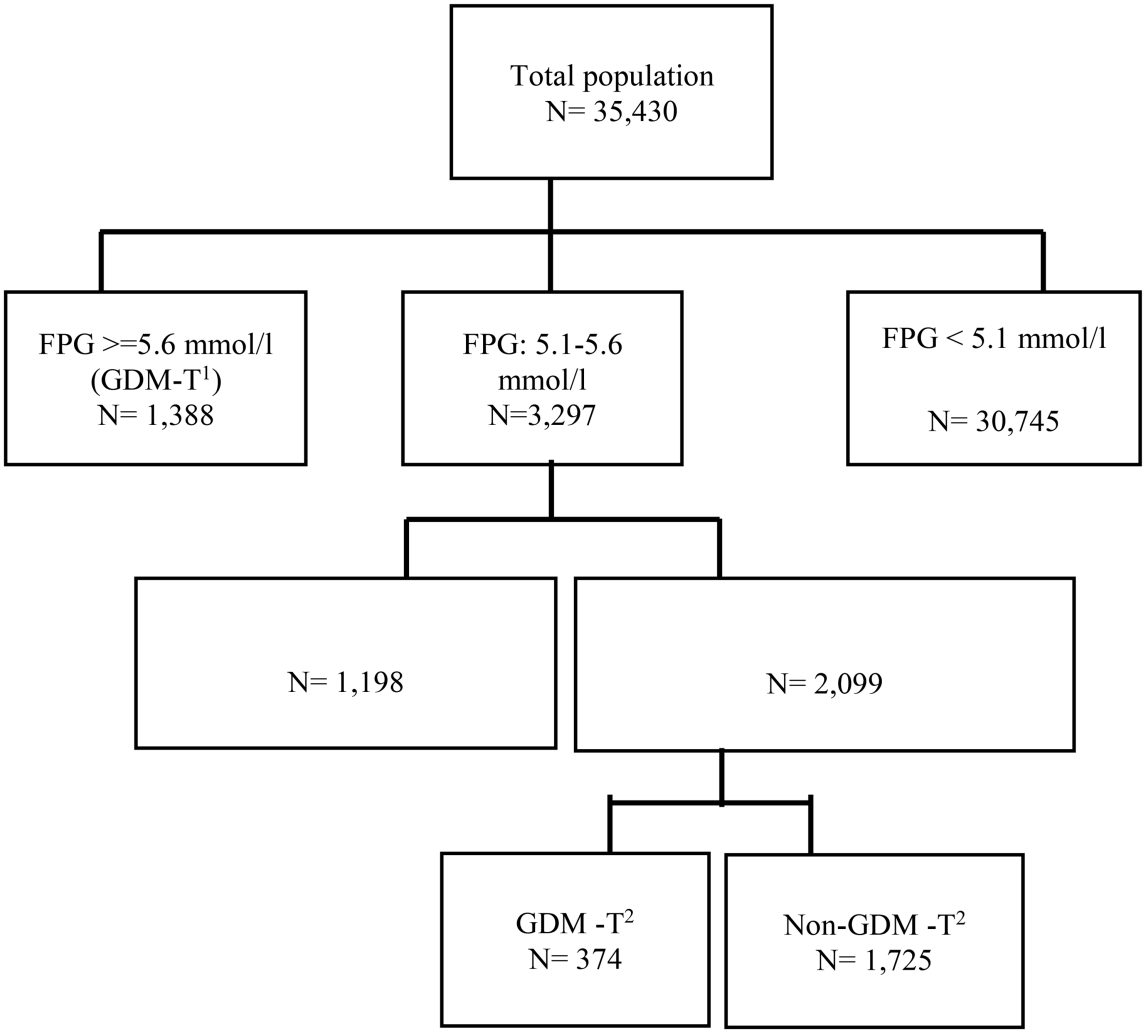
Flowchart of the study. T^1^: first trimester, T^2^: second trimester, FPG: fasting plasma glucose. Intervention group: those who had FPG values range 5.1-5.6 mmol/l in the first trimester of gestation and received treatment for GDM along with usual prenatal care, Control group: those who had FPG values range 5.1-5.6 mmol/l in the first trimester of gestation and received usual-prenatal-care.

All of study participants were followed-up until delivery, and their outcomes were registered in detail. The guidelines recommended by the American College of Obstetricians and Gynecologists (ACOG) 2013 (21) and the American Diabetes Association (ADA) 2016 (22) were used as the basis for the treatment of GDM, which included physical exercise, dietary intervention, and pharmacological therapy if necessary. The flowchart of the current study is presented in **Figure 1**.

### Endpoint outcomes

Macrosomia/large for gestational age (LGA) and primary cesarean section (C-S) were considered primary outcomes. Secondary outcomes were preterm birth before 37 weeks of gestation, admission to the neonatal intensive care unit (NICU), neonatal hypoglycemia, neonatal hypocalcemia, neonatal hyperbilirubinemia, preeclampsia, birth trauma, and low birth weight (LBW).

Macrosomia was defined as birth weight> 4000 g (23) and primary cesarean section was defined as the cesarean deliveries out of all births to women who had not had a previous cesarean. Definition of other outcomes had been published elsewhere (14, 19, 24–26).

### Statistical analysis

The present study had a power of 95% to detect 40% increased risk in macrosomia outcome based on their mild GDM treatment status in the first of pregnancy, with a two-sided 5% significance level, and a sample size of 3,297 participants. Continuous variables were checked for normality using the Shapiro–Wilk test. Characteristics of participants were compared using the independent t-test or Pearson’s Chi-squared test for continuous and categorical data, and also Mann–Whitney U test for variables with skewed distribution. A modified Poisson regression for binary outcome data with a log link function and robust error variance was used to estimate relative risks (RRs) and 95% confidence intervals (CIs) for the associations between mild GDM treatment status at the first trimester (treated mild GDM and non- treated mild GDM) and incidence of pregnancy outcomes; For comparison of first trimester treated mild GDM with those subgroup of first trimester non- treated mild GDM who developed GDM in the second trimester, adjusted risk ratios and 95% CI were reported. Adjusted variables were gestational ages at entrance and delivery, maternal BMI, gestational weight gain, method of GDM screening in the second trimester, and type of delivery. Treatment modality was also adjusted to compare those subgroups of GDM diagnosed in the second and first trimester of pregnancy treated mild GDM. Moreover, we adjust type of test in the model for comparing subgroup of first trimester non- treated mild GDM who developed GDM in the 2nd trimester with those first trimester treated mild GDM. Both unadjusted and adjusted models were fitted. In all analyses related to primary cesarean section outcomes, those with a previous history of cesarean section were excluded. Penalized maximum likelihood estimation was applied in the case of sparse data. Since the study was a cluster randomized trial, the cluster effect was considered in the analysis and the significance level of the test was set as 0.025 for considering subgroup analyses (27). Finally, the plot of the relative risk was depicted for all pregnancy outcomes by GDM treatment status. Statistical analysis was performed using STATA (version 13; STATA Inc., College station, TX, USA).

## Results

**Table 1** shows the characteristics of study participants according to study groups. The mean (SD) pregnancy week for the first prenatal visit in intervention and control groups were 8.2 (3.3) and 9.1 (3.3) weeks, respectively. The mean maternal age and BMI of pregnant women in intervention and control groups were similar (31.1 (6.1) vs. 30.7 (5.8) years, P-value = 0.082) and (26.9 (4.8) vs. 26.7 (4.8) kg/m^2^, P-value = 0.264), respectively. Compared to the control group, women with treated GDM in intervention group had a significantly lower gestational age at enrollment (8.2 (3.3) vs. 9.1 (3.3) weeks, P-value = 0.001), a slightly lower gestational age at delivery (38.6 (1.7) vs. 38.8 (1.7) weeks, P-value = 0.025), a significantly lower number of miscarriages (0.3 (0.6) vs. 0.5 (0.7), P-value = 0.001), and a slightly higher systolic blood pressure (102.9 (9.7) vs. 101.8 (9.5) mmHg, P-value = 0.004), although those differences may not carry clinical significance.

**Table 1:** Characteristics of study participants according to study groups.

Characteristics	Intervention* n = 1198	Controls** n = 2099	p-value
Background characteristics			
Age, year	31.1 (6.1)	30.7 (5.8)	0.082
BMI at first trimester, kg/m^2^	26.9 (4.8)	26.7 (4.8)	0.264
Gestational age at enrollment, week	8.2 (3.3)	9.1 (3.3)	**0.001**
Gestational age at delivery, week	38.6 (1.7)	38.8 (1.7)	**0.025**
Educational level, n (%)			
Elementary School	8 (0.7)	5 (0.2)	0.029
High school or Diploma	9 (0.7)	3 (0.1)	**0.003**
College degree	1 (0.08)	3 (0.1)	0.874
Gravity	2.3 (1.2)	2.2 (1.2)	0.315
Parity	1.1 (1.0)	1.0 (0.9)	0.846
Parity ≥1, n(%)	631 (52.7)	1332 (63.5)	0.035
Number of miscarriages^¥^	0.3 (0.6)	0.5 (0.7)	**0.001**
Systolic Blood Pressure, mmHg	102.9 (9.7)	101.8 (9.5)	**0.004**
Diastolic Blood Pressure, mmHg	64.7 (7.7)	63.8 (7.4)	**0.002**
Past history of adverse pregnancy outcomes^€^			
Gestational hypertension/preeclampsia, n (%)	21 (1.7)	30 (1.4)	0.516
Macrosomia, n (%)	17 (1.4)	46 (2.2)	0.457
Preterm birth, n (%)	24 (2.0)	44 (2.1)	0.532
Low Birth Weight, n (%)	25 (2.1)	60 (2.9)	0.745
GDM, n (%)	33 (2.7)	66 (3.1)	0.786
3^rd^ trimester vaginal bleeding, n (%)	7 (0.6)	5 (0.2)	0.041
Sever hemorrhage after delivery, n (%)	3 (0.2)	3 (0.1)	0.312
Fetal anomalies, n (%)	5 (0.4)	21 (1.0)	0.263
Twin pregnancy, n (%)	9 (0.7)	17 (0.8)	0.757
Stillbirth, n (%)	9 (0.7)	27 (1.3)	0.496
Instrumental delivery, n (%)	2 (0.2)	4 (0.2)	0.901
Family history			
Type 2 diabetes Mellitus, n (%)	119 (9.9)	277 (13.2)	**0.001**
Chronic hypertension, n (%)	135 (11.3)	356 (17.0)	0.047

^¥^It was defined as the loss of a pregnancy before the 20th week of gestation without any intentional intervention to end the pregnancy.

^€^Calculated for women with at least one parity;

Comparing GDM with Non-GDM;

Values are presented in Mean (SD) and Number (percentage) as appropriate;

Bold values indicate statistical significance;

BMI, Body mass index, GDM, gestational diabetes mellitus.

*Intervention group: those who had FPG values range 5.1-5.6 mmol/l in the first trimester of gestation and received treatment for GDM along with usual prenatal care (n=1,198),

**Control group: those who had FPG values range 5.1-5.6 mmol/l in the first trimester of gestation and received usual-prenatal-care (n=2,099).

**Figure 2:**
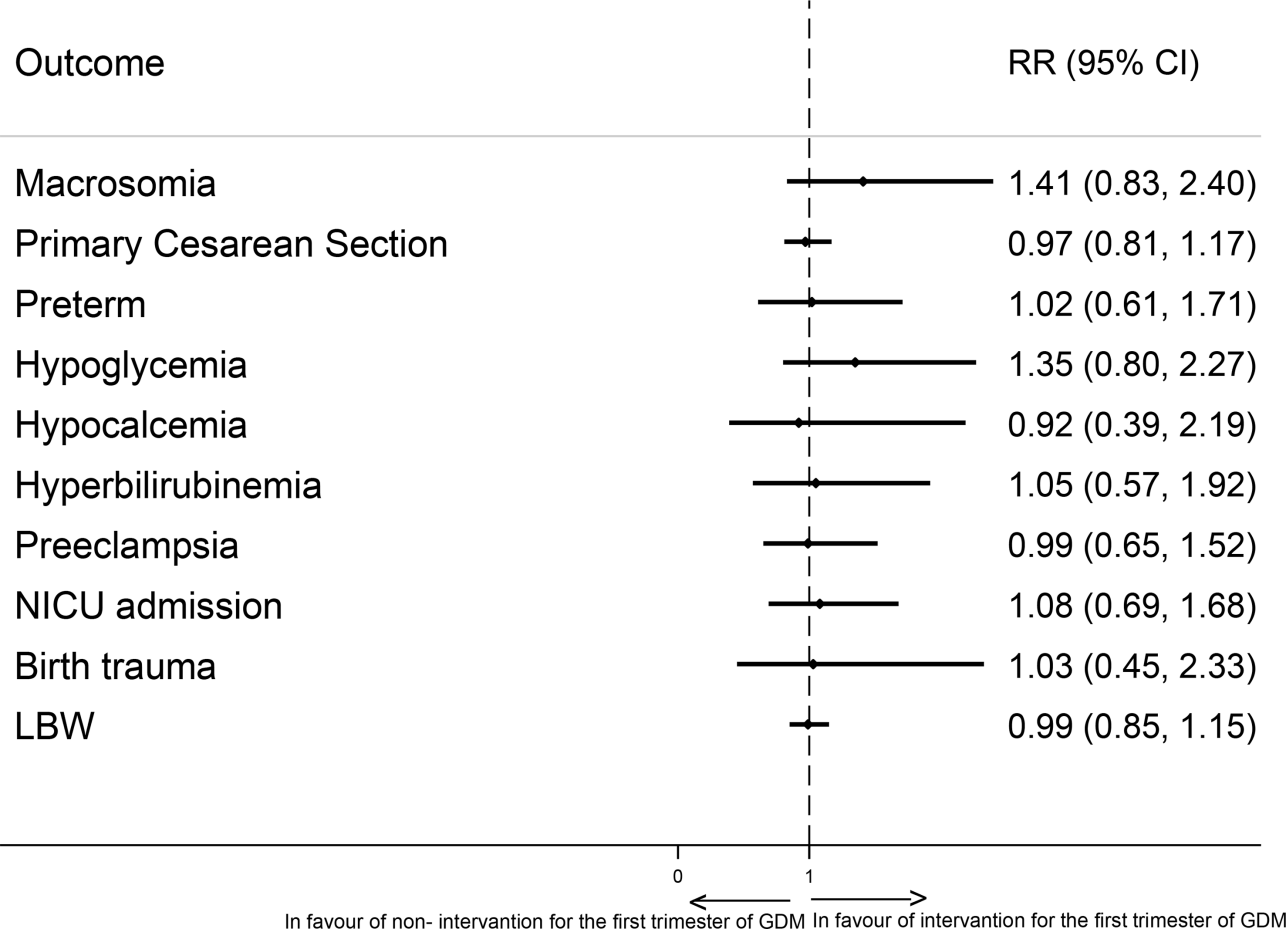
Adjusted risk ratio plot for pregnancy outcomes comparing intervention group and controls.

The prevalence and Risk ratio (95% CI) of maternal and neonatal outcomes in pregnant women based on their GDM treatment status in the first of pregnancy are presented in **Table 2**. There was no statistically significant difference between groups in the frequency of the adverse pregnancy outcomes of macrosomia, primary C-S, preterm birth, neonatal hyperbilirubinemia, neonatal hypoglycemia, neonatal hypocalcemia, preeclampsia, NICU admission, birth trauma, and LBW considering multiplicity adjustment. There were no statistically significant differences in the adjusted risks of adverse pregnancy outcomes in intervention group compared to controls considering multiplicity adjustment (**Figure 2** and **Table 2**).

**Table 2:** Prevalence and Risk ratio and 95% confidence interval of adverse pregnancy outcomes in participants based on study groups§.

	Prevalence		P-value *	Unadjusted Model		Adjusted Model ^§^	
	GDM (n = 1198)	Non-GDM (n = 2099)		RR (95% CI)	P-value *	RR (95% CI)	P-value **
Macrosomia	89 (7.8)	124 (6.4)	0.1	1.23 (0.74-2.01)	0.4	1.41 (0.83-2.40)	0.2
Primary cesarean-section ^¥^	175 (19.8)	294 (21.0)	0.5	0.94 (0.74-1.19)	0.6	0.97 (0.81-1.17)	0.8
Preterm birth ^§^	79 (6.9)	131 (6.7)	0.8	1.03 (0.64-1.66)	0.9	1.02 (0.61-1.71)	0.9
Neonatal Hypoglycemia	30 (2.6)	29 (1.5)	0.03	1.77 (1.11-2.80)	**0.015**	1.35 (0.80-2.27)	0.2
Neonatal Hypocalcemia	19 (1.6)	22 (1.0)	0.2	1.51 (0.77-3.00)	0.2	0.92 (0.39-2.19)	0.9
Neonatal Hyperbilirubinemia	96 (8.5)	130 (6.8)	0.08	1.26 (0.58-2.74)	0.5	1.05 (0.57-1.92)	0.8
Preeclampsia	124 (10.3)	218 (10.4)	0.9	1.00 (0.60-1.65)	0.9	0.99 (0.65-1.52)	0.9
NICU admission	82 (6.8)	112 (5.3)	0.08	1.29 (0.82-2.01)	0.3	1.08 (0.69-1.68)	0.7
Birth trauma	9 (0.7)	14 (0.7)	0.8	1.13 (0.37-3.48)	0.8	1.03 (0.45-2.33)	0.9
Low Birth Weight ^€^	94 (8.4)	162 (8.5)	0.9	0.99 (0.77-1.27)	0.9	0.99 (0.85-1.15)	0.9

Bold values indicate statistical significance, Significance level was set as 0.025 for considering multiple comparisons.

GDM, gestational diabetes mellitus; NICU, neonatal intensive care unit; CI, confidence interval.

*Significance level was set as 0.05.

**Significance level was set as 0.025 considering multiple comparisons.

^§^Adjusted variables were gestational age at enrollment and delivery, maternal BMI, gestational weight gain, and type of delivery; ^¥^ For outcome of primary cesarean-section women with repeated C-section were excluded, ^€^ For outcome of LBW women with abortion were excluded, ^§^ For outcome of preterm birth, gestational age at delivery was not adjusted. Bold values indicate statistical significance, Significance level was set as 0.025 for considering multiple comparisons. RR: risk ratio, NICU: neonatal intensive care unit; IUFD: Intrauterine fetal death;

§Intervention group: those who had FPG values range 5.1-5.6 mmol/l in the first trimester of gestation and received treatment for GDM along with usual prenatal care, Control group: those who had FPG values range 5.1-5.6 mmol/l in the first trimester of gestation and received usual-prenatal-care.

Reference group is control group.

The prevalence and Risk ratio (95% CI) of maternal and neonatal outcomes in pregnant women with first trimester treated GDM in intervention group (n=1198) compared to those subgroup of control group who developed GDM in the second trimester (n=374) are presented in **Supplementary Table 1** (**Table S1**). The prevalence of maternal and neonatal outcomes, except for neonatal hypoglycemia and hypocalcemia, were similar in intervention group compared to those subgroup of controls who developed GDM in the second trimester; the frequency of neonatal hypoglycemia and hypocalcemia in the latter group were significantly higher than first trimester intervention group (6.8% vs. 2.6%, P-value < 0.001 and 4.5% vs. 1.6%, P-value = 0.001, respectively). There were no statistically significant differences in the adjusted risks of adverse pregnancy outcomes in those groups, considering multiplicity adjustment, except for hypocalcemia (RR = 2.05; 95% CI: (1.12, 3.75); P = 0.02) (**Table S1** and **Figure S1**).

## Discussion

In this secondary analysis of a randomized community non-inferiority trial involving pregnant women with FPG level between 5.1-5.6 mmol/l in the first trimester of pregnancy, we found that GDM treatment at the first trimester of pregnancy was not associated with a reduced risk of adverse maternal and neonatal outcomes, including macrosomia, LBW, primary C-S, preterm birth, neonatal hypoglycemia, neonatal hypocalcemia, hyperbilirubinemia, preeclampsia, NICU admission, and birth trauma. Moreover, the adjusted risks of adverse pregnancy outcomes in pregnant women with FPG between 5.1-5.6 mmol/l who did not receive GDM care but developed GDM in the second trimester were not significantly different from those with FPG between 5.1-5.6 mmol/l at first trimester who received GDM cares.

There is ongoing debate regarding the benefits and risks of screening for and treating hyperglycemia in the first trimester of pregnancy (3, 28) Most current guidelines for GDM screening are based on studies initiating diagnosis and subsequent treatment in the third trimester (6, 29, 30). The evidence available is insufficient to determine the balance of benefits and harms of early screening for and treating GDM (4). Accordingly, studies that focus on the potential benefits of early GDM screening have yielded mixed and conflicting results (31–34). Whereas some studies have reported an association between GDM and maternal and neonatal morbidity (35–37), it has also been demonstrated that early treatment for GDM does not substantially improve these potential adverse pregnancy outcomes (13, 38, 39). Additionally, recent data have failed to demonstrate a clear association between FPG levels in early pregnancy and later GDM at 24–28 weeks of gestation (8, 9). It is important to note that most of the available data were derived from retrospective studies, and there is a lack of randomized controlled trials, which could potentially provide more robust evidence.

Our community randomized trial results showed that early diagnosis and treatment of GDM did not improve pregnancy outcomes. However, there are few randomized trial to compare early versus routine screening for GDM. In agreement with our findings, Osmundson et al. (2016), conducted an RCT, to assess the effect of early GDM treatment prior to 14 weeks gestation in women with a hemoglobin A1c (Hb A1c) levels between 5.7–6.4%. The study randomly assigned 42 pregnant women to receive GDM treatment and 41 to receive usual prenatal care. The results showed that early treatment for women with prediabetes-range A1C levels in the first-trimester did not significantly reduce the risk of GDM diagnosis by the second trimester (40).

In another RCT, Roeder et al. (2019) assessed whether treating women with mild glucose intolerance earlier in pregnancy would be beneficial in the reduction of adverse perinatal outcomes. Pregnant women with hyperglycemia, including Hb A1c ≥5.7% and/or FPG ≥5.1 mmol/l in early pregnancy were randomized to early pregnancy or third-trimester treatment of GDM. Results of the study showed that treatment in early pregnancy did not significantly improve maternal or neonatal outcomes, including macrosomia, weight for length percentile at birth, maternal gestational weight gain, fat mass, and cord blood C-peptide > 90th percentile (41). In a recently published RCT, Harper et al. (2020) conducted an RCT to assess whether early screening for GDM improves perinatal outcomes in a high-risk group of obese pregnant women with BMI ≥ 30 kg/m^2^. The study involved 922 pregnant women randomized to early (n= 459) and routine (n= 463) GDM screening. The researchers reported that early screening for GDM in obese women did not reduce the composite perinatal outcome that included any of the following outcomes of macrosomia, primary cesarean delivery, pregnancy-induced hypertension, shoulder dystocia, neonatal hypoglycemia and neonatal hyperbilirubinemia (38). In a secondary analysis of data from the lifestyle in pregnancy study, Vinter et al. (2018), assessed a lifestyle intervention among obese women fulfilling the GDM WHO-2013 diagnostic criteria in early gestation. In this trial, 36 participants received lifestyle intervention and 54 received standard care. The researchers found that all metabolic parameters and obstetric outcomes were similar in both groups. However, there were more planned C-S in the lifestyle intervention group (42). Finally, preliminary results of a randomized controlled trial in Australia (TOBOGM study) among singleton high-risk pregnant women showed that treatment for GDM in early gestation might have both benefits and harms (13). The study found that early GDM treatment is associated with a reduced LGA rate but an increased NICU admission rate mainly due to an increase in small for gestational age (SGA) that may be resulted from fetal undernutrition as a consequence of overtreatment, or insufficient gestational weight gain, with putative long term consequences.

Our study revealed that an FPG of 5.1 mmol/l may not be considered an optimal glycemic target early in pregnancy; it was not associated with an improvement in pregnancy outcomes, and 82% (1725/2099) those pregnant women who had FPG values 5.1-5.6 mmol/l and did not receive treatment for GDM in the first trimester, did not meet the GDM criteria at second trimester. These results are consistent with those of a study conducted among Chinese subjects, which reported a 37% incidence of GDM for women with FPG values between 5.1 and 5.6 mmol/l at the first prenatal visit (8). Adopting this threshold for GDM diagnosis may overload the healthcare system and create stress and psychological burden for pregnant women impacting their quality of life and overall well-being during pregnancy (17). Labeling pregnant women with a cut-off value of 5.1 mmol/l for FPG early in pregnancy as GDM did not result in any improvements in maternal or neonatal outcomes; Instead, it has led to the over-medicalization of pregnancy, and healthcare costs in many hitherto healthy pregnant women that will be labeled as GDM. While the first-trimester FPG value is important for the diagnosis of pregestational overt diabetes, a value of 5.1 mmol/l is a poor predictor for GDM early in pregnancy and may lead to false-positive results. Therefore, treatment of this group of pregnant women early in pregnancy does not translate into improved outcomes.

Our study has several strengths, including community-based trial design of study, large sample size, geographic distribution of the regions involved, broad inclusion criteria and using similar laboratory protocols. Unlike many previous studies, which focused on high-risk populations, our study evaluated a general population of pregnant women. We also adjusted for potential risk factors, which adds to the strength of our findings. Additionally, the early enrollment of participants, on average at eight weeks of gestation, is a further strength of our study. However, there are some limitations to our trial that should be acknowledged. According to the Iranian national guidelines for prenatal care, pregnant women with known chronic disorders should be directly referred to the second level of the healthcare system and receive their prenatal care there, rather than in a primary healthcare setting, which was the platform of our study. Evaluating the adverse feto-maternal and neonatal outcomes in these high-risk groups was beyond our research aim of study. As a result, our findings are not generalizable to those with various chronic disorders. Moreover, we did not measure HbA1c in our study. Additionally, we did not used a central reference laboratory for measurements. However, all of procedures, equipment, and supplies were homogeneous in all laboratory sites, and monthly external quality-controls were done for each laboratory. Our study was conducted among Iranian women; the results may not be applicable to other populations.

In conclusion, our study suggests that using the first-trimester FPG values of 5.1-5.6 mmol/l as the criteria to diagnose GDM is not recommended. Treatment of these women did not lead to improved adverse pregnancy outcomes, and they may not require the special prenatal care recommended for those with a GDM diagnosis. Therefore, we recommend that this group of pregnant women be re-screened for GDM at 24-28 weeks of gestation.

## Data availability statement

The raw data supporting the conclusions of this article will be made available by the authors, without undue reservation.

## Ethics statement

National Institute for Medical Research Development under Grant Agreement No IR.NIMAD.REC.1394.013 has been approved and funded the study (but did not involve in the study). In addition, the national ethics committee of the National Institute for Medical Research Development approved the study protocol (Approval number: IR.NIMAD.REC.1394.013) and the Iranian Ministry of Health and Medical Education (MoHME) approved the study protocol. The pre-specified GDM modalities were made available to all those provinces as mandatory guidelines. Written informed consent for participation was not required for this study in accordance with the national legislation and the institutional requirements.

## Author contributions

FRT, SB-G, and FA conceived and designed the trial and FRT is the chief investigator. FFa, FFi, FHo, MAb, FHa, MV, FT, DK, MS-D, MB, AO, MAm, and FA contributed to the protocol and design of the study. RB-Y, MR, DK, and MS-D did the statistical analysis. FRT, SB-G, FHo, FFi, FFa and MR contributed to the preparation of the report. All authors contributed to the implementation of the study and data collection. All authors contributed to the article and approved the submitted version.
