# Supplementary material for: Does fasting plasma glucose values 5.1-5.6 mmol/l in the first trimester of gestation a matter?
Source: Front Endocrinol (Lausanne). 2023 Jun 2;14:1155007. doi: 10.3389/fendo.2023.1155007 (PMC10273274; doi:10.3389/fendo.2023.1155007)
Supplement: Supplementary file 1 [file DataSheet_1.docx]

**Supplementary file**

**Supplementary Table 1.** Prevalence and Risk ratio (RR) and 95% CI for adverse pregnancy outcomes comparing GDM-T^2^ (n=374) and intervention group (n=1198).

| Outcomes | Prevalence | |  | Unadjusted | | Adjusted | |
| --- | --- | --- | --- | --- | --- | --- | --- |
|  | GDM-T^2^  n = 374 | GDM-T^1^  n = 1198 | P-value* | RR (95% CI) | P-value* | RR (95% CI) | P-value** |
| Macrosomia | 19 (5.4) | 89 (7.8) | 0.1 | 0.69 (0.38-1.25) | 0.2 | 0.46 (0.23-0.95) | 0.04 |
| Primary cesarean-section ^¥^ | 46 (18.6) | 175 (19.8) | 0.7 | 0.94 (0.73-1.22) | 0.7 | 0.80 (0.59-1.07) | 0.1 |
| Preterm birth ^§^ | 27 (7.6) | 79 (6.9) | 0.6 | 1.10 (0.64-1.90) | 0.7 | 0.75 (0.42-1.37) | 0.3 |
| Neonatal Hypoglycemia | 24 (6.8) | 30 (2.6) | **<0.001** | 2.57 (1.76-3.76) | **<0.001** | 1.63 (0.98-2.71) | 0.06 |
| Neonatal Hypocalcemia | 17 (4.5) | 19 (1.6) | **0.001** | 2.86 (1.45-5.62) | **0.002** | 2.05 (1.12-3.75) | **0.02** |
| Neonatal Hyperbilirubinemia | 29 (8.2) | 96 (8.5) | 0.8 | 0.96 (0.39-2.35) | 0.9 | 0.77 (0.36-1.67) | 0.5 |
| Preeclampsia | 44 (11.8) | 124 (10.3) | 0.4 | 1.13 (0.60-2.15) | 0.5 | 0.96 (0.55-1.66) | 0.9 |
| NICU admission | 29 (7.7) | 82 (6.8) | 0.5 | 1.13 (0.60-2.15) | 0.7 | 1.03 (0.83-1.29) | 0.8 |
| Birth trauma | 5 (1.3) | 9 (0.7) | 0.3 | 1.77 (0.62-5.08) | 0.3 | 1.91 (0.64-5.73) | 0.2 |
| Low Birth Weight ^€^ | 34 (9.6) | 94 (8.4) | 0.4 | 1.15 (0.92-1.44) | 0.2 | 0.97 (0.76-1.23) | 0.7 |
| * Significance level was set as 0.05.  ** Significance level was set as 0.025 considering multiple comparisons  Adjusted variables were gestational age at enrollment and delivery, maternal BMI, gestational weight gain, type of delivery and type of test; ^¥^ For outcome of primary cesarean-section women with repeated C-section were excluded, ^€^ For outcome of LBW women with abortion were excluded, ^§^ For outcome of preterm birth, gestational age at delivery was not adjusted. Bold values indicate statistical significance, Significance level was set as 0.025 for considering multiple comparisons.  RR: risk ratio, NICU: neonatal intensive care unit;  Intervention group: those who had FPG values range 5.1-5.6 mmol/l in the first trimester of gestation and received treatment for GDM along with usual prenatal care, GDM-T^2^: pregnant women with FPG values range 5.1-5.6 mmol/l in the first trimester of gestation who received usual prenatal care (control group), but developed GDM at 2nd trimester.  Reference group is intervention group. | | | | | | | |

**Supplementary Figure 1**. Adjusted risk ratio plot for pregnancy outcomes comparing GDM-T^2^ (n=374) and intervention group (n=1198).

Intervention group: those who had FPG values range 5.1-5.6 mmol/l in the first trimester of gestation and received treatment for GDM along with usual prenatal care, GDM-T^2^: pregnant women with FPG values range 5.1-5.6 mmol/l in the first trimester of gestation who received usual prenatal care (control group), but developed GDM at 2nd trimester.

Reference group is intervention group.
